# Composition and content analysis of fluoride in inorganic salts of the integument of Antarctic krill (*Euphausia superba*)

**DOI:** 10.1038/s41598-019-44337-6

**Published:** 2019-05-27

**Authors:** Yuanhuai Peng, Wei Ji, Di Zhang, Hongwu Ji, Shucheng Liu

**Affiliations:** 1 College of Food Science and Technology, Guangdong Ocean University, Guangdong Provincial Key Laboratory of Aquatic Products Processing and Safety, Key Laboratory of Advanced Processing of Aquatic Products of Guangdong Higher Education Institution, 524088 Zhanjiang, P.R. China; 2 College of Biological and Food Engineering, Guangdong University of Education, 510303 Guangzhou, P.R. China; 3 School of Chemistry and Chemical Engineering, Lingnan Normal University, 524048 Zhanjiang, P.R. China

**Keywords:** Bioinorganic chemistry, Biophysical chemistry

## Abstract

Ash of Antarctic krill integument (AAKI) was prepared by sintering the integument at 550**°**C under air atmosphere for 4 hours, and its composition was analyzed by X-ray diffraction (XRD), fourier transform infrared spectroscopy (FTIR) and electron dispersive spectroscopy (EDS). XRD results showed that the major phase in AAKI was ascribed to apatite. Besides, it was noticed that the (300) peak of AAKI shifted to 33.07°, which was coincident with that of fluorapatite (FA). The FTIR results confirmed the presence of phosphate ions, and the absence of -OH. The EDS results confirmed the presence of Ca, P, O and F elements in the ash sample. The content of FA in the ash was determined to be 50.4%, and the proportion of fluorine in the form of FA to the total fluorine in the integument was 40.5%. Based on the XRD, FTIR and EDS results, it can be concluded that FA was the main form of fluoride in the integument of Antarctic krill.

## Introduction

Antarctic krill has an abundant standing stock and represents a resource for human use. It has been found that whole krill contains 77.9–83.1% moisture, 0.4–3.6% lipids, 11.9–15.4% protein, and approximately 2.0% chitin^1^. Krill protein is considered to be of high quality, meeting FAO/WHO requirements for human consumption^2^. Apart from egg protein, the biological value of krill protein is higher than that of milk or other animal proteins^3^. Its effective utilization could help to alleviate the ever-increasing demand for food. High levels of fluoride have been found in Antarctic krill protein^4^. Ingestion of 1.7 μg/mL fluoride (Expression in F content) in drinking water can cause mottling of the teeth in 30–50% of humans, and chronic fluorosis may cause osteosclerosis, calcification of ligaments and tendons, bony exostoses, and renal calculi^5^.

High fluoride is one of the key issues restricting the exploitation and utilization of Antarctic krill. It has been found that fluoride is almost exclusively fixed in the integument of Antarctic krill, with 2594 ppm (F content, dry weight) reported in the integument, but only 6 ppm in the soft tissues^6^. Fluoride moves from the integument into the soft tissues where it is continuously accumulated in postmortem storage^7^. As high as 570 ppm fluoride have been found in the muscle of preserved krill^8^.

The migration of fluoride from the integument to the muscle leads to high levels of fluoride in the muscle. The cause of this migration is unknown, but an investigation into the form of fluoride in the integument would help understanding this issue. The newly formed integument is extremely thin (<5 μm) and soft, with a fluoride content lower than 100ppm. Fluoride contents in the integument are low in the case of post-molt, but increase rapidly during the early stages of the molting cycle^6^. This stage is characteristic of the structural completion and general consolidation of the new integument after molting. The integument is hardened due to sclerotization as well as calcification^9^. The increase in fluoride concentration is related to the consolidation of the new krill cuticle. Fluoride is probably in the form of FA which acted as a hardening agent, arising from the related distributions of the major elements of fluorine, calcium and phosphorous in the integument^10^. However, studies relative to the composition of the inorganic salts in the integument are still seldom, which could not provide additional data to support this speculation. Therefore, the objective of this study is to analyze the main form of fluoride in the integument of Antarctic krill and to provide basic knowledge of fluoride migration in postmortem preservation.

## Results

### Thermogravimetric analysis

**Figure 1:**
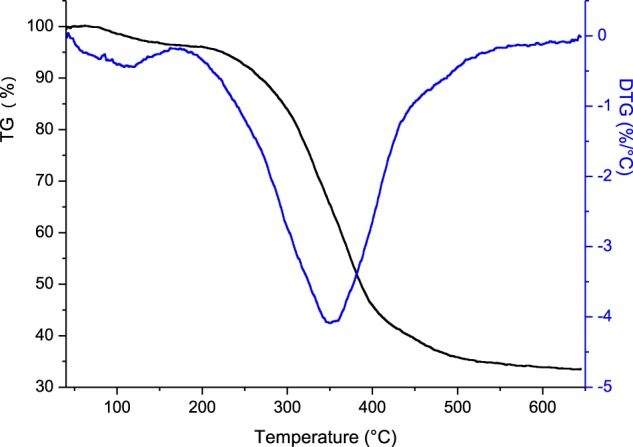
TGA diagram of Antarctic krill integument.

The thermogravimetry and differential thermogravimetry curves of the krill integument are presented in Fig. 1. Three degradation stages can be observed. The first stage was the dehydration process, in which absorbed water was evaporated before 150 °C. The second stage was regarded as the active pyrolysis process. During this stage, feedstock degradation and volatile release were prominent. The final stage was the positive pyrolysis process, which involves the re-decomposition and carbonization of the char residue. No weight loss was detected from 500 to 650 °C, which indicated that there was no decomposition of inorganic salts at this stage.

### X-ray diffraction

**Figure 2:**
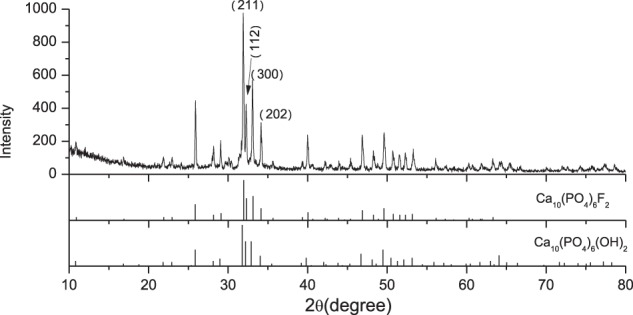
XRD of the ash of Antarctic krill integument.

XRD results of the ash from the integument of Antarctic krill are shown in Fig. 2. The XRD patterns were attributed to the apatite pattern (Ca_10_(PO_4_)_6_(OH)_2_, HA, JCPDS card number 09–0432; Ca_10_(PO_4_)_6_F_2_, FA, JCPDS card number 15-0876). As the XRD patterns of HA were similar to those of FA, it was hard to distinguish them.

### Fourier transform infrared spectroscopy

**Figure 3:**
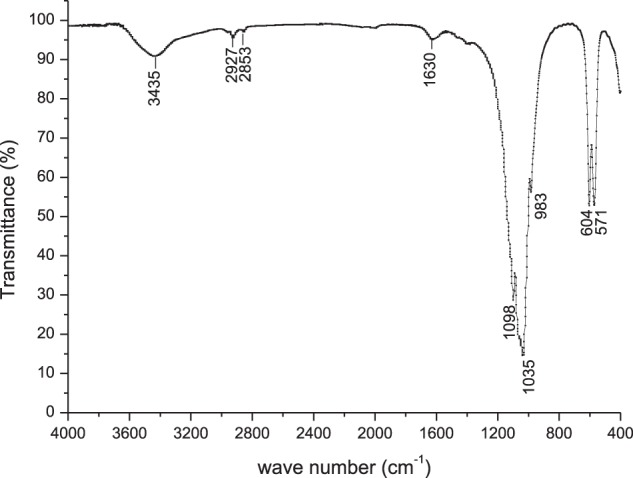
FTIR spectrum of the ash of Antarctic krill integument.

The FTIR results of AAKI are shown in Fig. 3. The peaks at 571, 604, 983, 1035, and 1098 cm^−1^ are associated with the vibrational modes of the phosphate groups. The peak located at 3435 cm^−1^ and the weak peak at 1630 cm^−1^ are ascribed to adsorbed water. The sharp peaks at 571 and 604 cm^−1^ were are indicative of the higher crystallinity of the apatite samples. The peaks situated at approximately 2853 and 2927 cm^−1^ are connected with small amounts of organic compounds which were not decomposed thoroughly.

### Electron dispersive spectroscopy analysis

**Figure 4:**
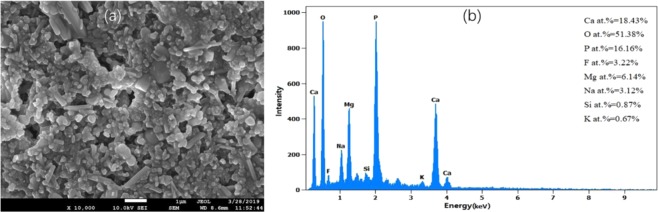
The SEM images of the ash of Antarctic krill integument after sintering at 550 °C under air atmosphere for 4 h and EDS spectra: (**a**) the SEM images of the ash; (**b**) The EDS spectrum of Fig. 4a.

The elemental composition of the ash was further characterized by EDS. The EDS spectrum (Fig. 4b) showed that the ash contained Ca, P, O and F elements.

### Total fluoride content in krill integument and fluoride content in the ash of krill integument

Total fluoride content of Antarctic krill integument was 3140 ± 86 ppm (d.w.) and fluoride content in the ash of Antarctic krill integument was 9547 ± 334 ppm.

## Discussion

The development and utilization of Antarctic krill has attracted enormous attention due to its high protein content. However, its utilization remained insufficient because of a high level of fluoride content. In order to make full use of this protein source, intensive research has been carried out to decrease fluoride content by chemical treatment^11^, prepare polypeptides^12^, extract protein isolates with low levels of fluoride^2^. Despite some advances, it is still hard to fully utilize this potential protein source. Basic theoretical research on Antarctic krill is still lacking. The form of fluoride presented in Antarctic krill and the reason for the migration of fluoride in postmortem Antarctic krill remain unknown. So, this study will be focused on determining the inorganic fluoride forms and their content in the integument of Antarctic krill.

In order to ascertain the form of inorganic fluoride in the integument of Antarctic krill, ash samples were attained by sintering the integument in an oven at 550 °C for 4 hours. The TGA results of Antarctic krill integument (Fig. 1) showed that only organic material was burned out and the decomposition of inorganic salts was not detected. Sintering condition guaranteed that the residuals only contained inorganic salts which were not decomposed during calcination process. Kaewtatip *et al*.^13^ reported that the weight loss at ~400 °C originated from the decomposition of organic components such as proteins and chitin of shrimp shell powder, and the decomposition of inorganic salts occurred at 688 °C. Poompradub *et al*.^14^ studied the degradation of cuttlebone. The results showed that weight loss from 650 to 825 °C was caused by the decomposition of inorganic salts.

The XRD results showed that the major phase of the ash was apatite. Dehydroxylation of HA took place at temperatures ranging from 850 to 900 °C^15^, and the initial fluorine emission temperature of FA is approximately 715 °C^16^. Combined with TGA results, it was clear that apatite was present in the integument. The slight up shift of the (300) peak toward a higher angle in FA compared with that of HA is related to α-axis contraction which arises from the substitution of smaller F atoms for -OH^17^. Hahn *et al*.^18^ investigated the effect of fluorine dopant on the performance of hydroxyapatite coating. Compared with the XRD patterns of FA, HA and synthetic powders, there was a slight shift of the (300) peak toward a higher angle with the increased fluoride content in the powders. The (300) peak of FA and HA were located at 33.13° and 32.90°, respectively. In our experiment, the (300) peak of the ash occured at 33.07°, coinciding with that of FA. The XRD results indicated that the major phase of the ash was FA.

The FTIR spectra of the ash showed typical absorption peaks of phosphate groups^19^. It was reported that the vibrational mode at 630 cm^−1^ and stretching mode at 3572 cm^−1^ of -OH were present in the HA coating but absent in the FA coating due to the substitution of F group for -OH^18,20^. In view of the lack of the -OH vibrational and stretching peaks in the ash of the integument, the main component of the ash could be determined to be FA. The FTIR analysis was consistent with the XRD results.

EDS spectra confirmed the existence of Ca, P, O, F along with other minor mental elements in the ash sample. Combined with the XRD, FTIR and EDS results, it can be concluded that FA exists in the integument of Antarctic krill. Zhang *et al*.^10^ analyzed the elements of the krill integument and speculated that Ca and P would readily combine with fluoride to produce FA, a known hardening agent. Sands *et al*.^21^ analyzed the F concentration in different parts of krill and found that krill mouthparts, the hardest part of the exoskeleton, contained the highest level of fluoride (12876 ppm), about six times higher than that of the whole exoskeleton, suggesting that F was in the form of FA so as to consolidate the integument.

Table 1 compared the fluoride content in the integument of Antarctic krill with those previously reported. A comparison of the results showed that fluoride levels recorded in the integument of Antarctic krill differed considerably. This may be due to differences in sample preparation methods and rapid autolysis in dead krill, leading to a decrease in protein content^22^. Meanwhile, migration of fluoride from the integument into the soft tissues would have an influence on fluoride content in the integument^7^.

**Table 1.** A comparison of fluoride content in Antarctic krill integument with published data.

Integument part	F content (ppm, d.w.)	Source
Integument	3140 ± 86	This work
Integument (Cephalothorax) Integument (abdomen)	1899 ± 470 2035 ± 724	ref.^21^
Integument	2594	ref.^6^
Integument	3330	ref.^8^
Integument	1958	ref.^31^

The ash content of Antarctic krill integument was 13.3%, which was consistent with the reports of Chen *et al*. (12.1%)^23^ and Song *et al*. (14.3%)^24^. Antarctic krill is a poorly calcified crustacean, the integument is relatively low in calcium content and the whole-body calcium level is also less than that of many decapods^25^. The ash from the krill integument contained a high level of fluoride (9547 ± 335 ppm). This result was obtained from a single analysis of batched ash samples, and there were no other reports in the literature for comparison. Only FA was detected in the ash, so the percentage of fluorine in the form of FA accounting for the total fluorine in Antarctic krill integument (40.5%) can be calculated. The results showed that FA was the main form of fluoride in the integument of Antarctic krill.

In summary, based on the TGA results of Antarctic krill integument, the ash sample was prepared by sintering under air atmosphere and was analyzed by XRD, FTIR and EDS. It can be concluded that FA existed in the integument of Antarctic krill and the proportion of fluorine in the form of FA to the total fluorine in the integument was 40.5%. FA was the main form of fluoride in the integument of Antarctic krill. The results of this study support the hypotheses that FA exists in the integument of Antarctic krill^10,21^. Future research should try to focus on identifying the factors leading to fluoride migration in the preservation of Antarctic krill.

## Materials and Methods

### Preparation of Antarctic krill integument samples

Antarctic krill (*Euphausia superba*) caught in Antarctic waters (approximately 63°06′–63°06′S, 58°51′–58°40′W) in March 2017, which were frozen and squeezed into blocks, were provided by CNFC Overseas Fisheries Co., Ltd. (Beijing, China). The blocks were transported to the laboratory at −18 °C. Upon arrival, they were stored at −80 °C. All chemical agents used in this study were of analytical grade.

To prepare the integument, frozen Antarctic krill was thawed at 4 °C and the integument and muscle were separated on ice. The adhering muscular tissue was carefully removed from the lateral parts of the fresh integument, which was then dried in an oven overnight at 80 °C.

The ash of the krill integument was prepared following AOAC methods^26^. Ash was obtained by burning the krill integument sample in a muffle furnace (SX-G02163, Tianjin Central Furnace Co., Ltd, China) at 550 °C for 4 hours under air atmosphere, at a heating rate of 10 °C /min, until a constant weight was obtained. The ash content was then determined.

### Thermogravimetric analysis

Thermogravimetric analysis (TGA) was carried out using a STA 449 thermogravimetric analyzer (Netzsch Wittelsbacherstr, Germany). High-purity nitrogen (99.99%) was used as the carrier gas with a flow rate of 20 mL/min. Approximately 2 mg of sample material was loaded in the ceramic crucible, and heated from 40 to 650 °C at a heating rate of 10 °C/min, and then maintained at the isothermal stage for 10 min.

### X-ray diffraction

The ash of krill integument was measured by X-ray diffractometry (XRD, X’Pert-MPD System, PHILIPS, Amsterdam, Netherlands). A continuous scan was performed using a step size of 0.01° and a step time of 0.2 s. All data were obtained using the MDI Jade software package (Jade 5.0, Materials Data, Inc., Japan)^27^.

### Fourier transform infrared spectroscopy

The Fourier transform infrared (FTIR) spectra of ash from the krill integument was measured over the 4000–400 cm^−1^ frequency range using 32 scans at a resolution of 4 cm^−1^ with a model TENSOR27 spectrometer (Bruker Corporation, Karlsruhe, Germany)^28^. Samples of ash were prepared into 0.25 mm thick KBr pellets (1 mg sample in 100 mg KBr) and stabilized under controlled humidity conditions, before determining the spectra. The curves which mathematically best fitted the original spectrum were obtained by invoking the Gaussian function using Origin Pro 8.5 software (Originlab Corporation, Northampton, USA)^29^. The intensity of the selected absorption band was determined by the baseline method on the basis of the OMNIC software package of the instrument.

### Electron dispersive spectroscopy analysis

A cold field emission scanning electron microscopy (S-4800, Hitachi, Tokyo, Japan) equipped with electron dispersive spectroscopy (EDS) was used to analyze the composition of elements in the ash. Samples were mounted on a conductive carbon imprint left by the adhesive tape prepared by placing the samples on the circular holder and coated for 5 min to enable conduction. Samples were analyzed by EDS at an accelerating voltage of 15 kV.

### Total fluoride content in krill integument and fluoride content in the ash of krill integument

Total fluoride concentration in the krill integument was analyzed following the method of Marian *et al*.^30^. To analyze the fluorine concentration in the ash, 10 mL of 1.0 mol/L HCl was added to the ash (0.1 g, weighed to the nearest 0.0001 g). This was left for 1 hour to extract the fluoride. The extracted solution was transferred to a 50-mL volumetric flask, then 25 mL TISAB (equal volume of 3.00 mol/L sodium acetate solution and 0.75 mol/L sodium citrate solution mixed for immediate use) was added and diluted to volume with distilled water.

Fluoride content was measured with a fluoride ion-selective electrode. The electrode was calibrated using a standard addition program. All fluoride levels were expressed in ppm (d.w.).

1 $${\rm{FA}} \% =\frac{F(a)\times {10}^{-6}\times 1004}{19.0}\times 100$$

Where FA% is the percentage of FA in the ash of Antarctic krill integument, F(a) is fluoride content in the ash of Antarctic krill integument, 10^−6^ is microgram and gram conversion coefficient, 1004 is molecular weight of FA and 19.0 is atomic weight of F.

2 $${\rm{S}} \% =\frac{{\rm{F}}({\rm{a}})\times {\rm{ash}} \% }{{\rm{F}}({\rm{t}})}\times 100$$

Where S% is the percentage of fluorine in the form of FA accounting for the total fluorine in Antarctic krill integument, F(a) is fluoride content in the ash of Antarctic krill integument, F(t) is fluoride content in Antarctic krill integument and ash% is ash content of Antarctic krill integument.

## Supplementary information

Supporting information for review
