# Supplementary material for: Composition and content analysis of fluoride in inorganic salts of the integument of Antarctic krill (Euphausia superba)
Source: Sci Rep. 2019 May 27;9:7853. doi: 10.1038/s41598-019-44337-6 (PMC6536536; doi:10.1038/s41598-019-44337-6)
Supplement: Supplementary file 1 — Supporting information for review [file 41598_2019_44337_MOESM1_ESM.docx]

**Composition and content analysis of fluoride in inorganic salts of the integument of Antarctic krill (*Euphausia superba*)**

Yuanhuai Peng^1,3^, Wei Ji^2^, Di Zhang^1^, Hongwu Ji^1^, Shucheng Liu^1^

^1^College of Food Science and Technology, Guangdong Ocean University, Guangdong Provincial Key Laboratory of Aquatic Products Processing and Safety, Key Laboratory of Advanced Processing of Aquatic Products of Guangdong Higher Education Institution, Zhanjiang 524088, P. R. China.

^2^College of Biological and Food Engineering, Guangdong University of Education, Guangzhou, 510303, P. R. China.

^3^School of Chemistry and Chemical Engineering, Lingnan Normal University, Zhanjiang, 524048, P. R. China.

Antarctic krill (*Euphausia superba*) has been attracted much attention due to its large standing stock and high quality of protein. High fluoride content has restricted its exploitation and utilization. During preservation, fluoride migrates from the integument to the muscle, leading to an increase in fluoride content which is beyond food safety regulations. During the process of fluoride migration, the existence of fluoride in the integument of Antarctic krill is bound to change. The analyzing of the composition of the inorganic salt in the integument would help understanding this phenomenon.

The work undertaken was the first time confirming that fluorapatite （FA, Ca_10_(PO_4_)_6_F_2_） exists in the integument of Antarctic krill by modern analytical methods and the proportion of fluorine in the form of FA to the total fluorine in the integument was 40.5%. The results of this study support the hypotheses that FA exists in the integument of Antarctic krill.
